# Supplementary material for: Brain network hypersensitivity underlies pain crises in sickle cell disease
Source: Sci Rep. 2024 Mar 27;14:7315. doi: 10.1038/s41598-024-57473-5 (PMC10973361; doi:10.1038/s41598-024-57473-5)
Supplement: Supplementary file 1 — Supplementary Information. [file 41598_2024_57473_MOESM1_ESM.pdf]

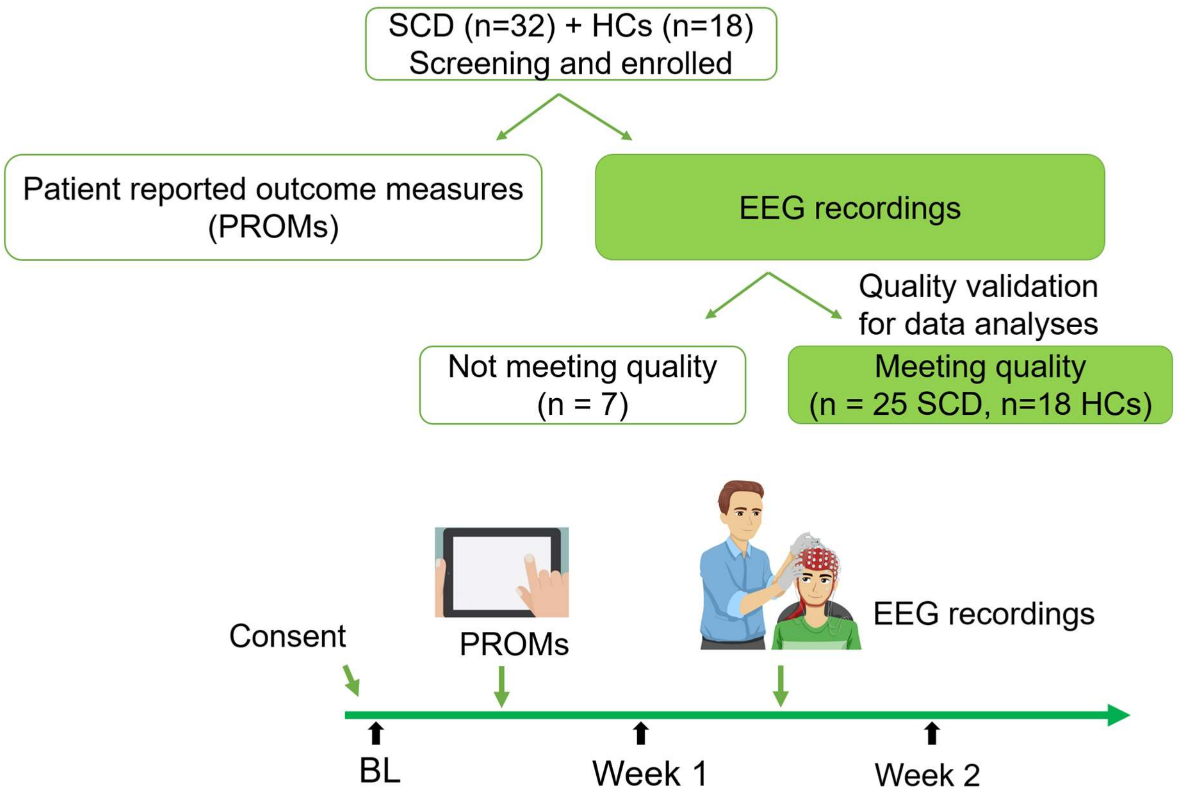

Supplementary Figure 1. Study workflow of enrollment and analyses

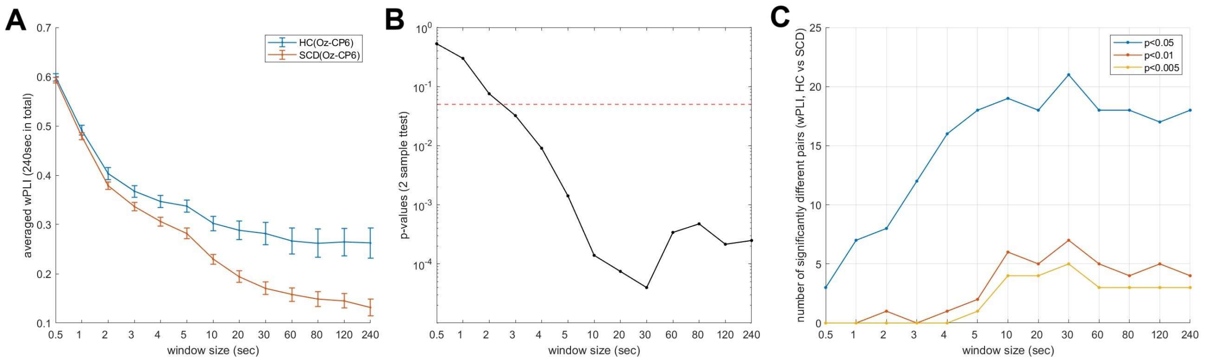

Supplementary Figure 2. Window size dependency of wPLI. A) wPLI values of HCs and SCD were compared across different window sizes. This pair of EEG channels were selected, which showed significant differences with the lowest p-values (Oz-CP6, p=0.00004) at the 30 second window. The error bar represents standard error. B) Corresponding p-values of two sample t-tests between HCs and SCD groups. The red horizontal line represents p=0.05. C) Number of significantly different wPLI pairs. 30 second window shows maximal number of significances.

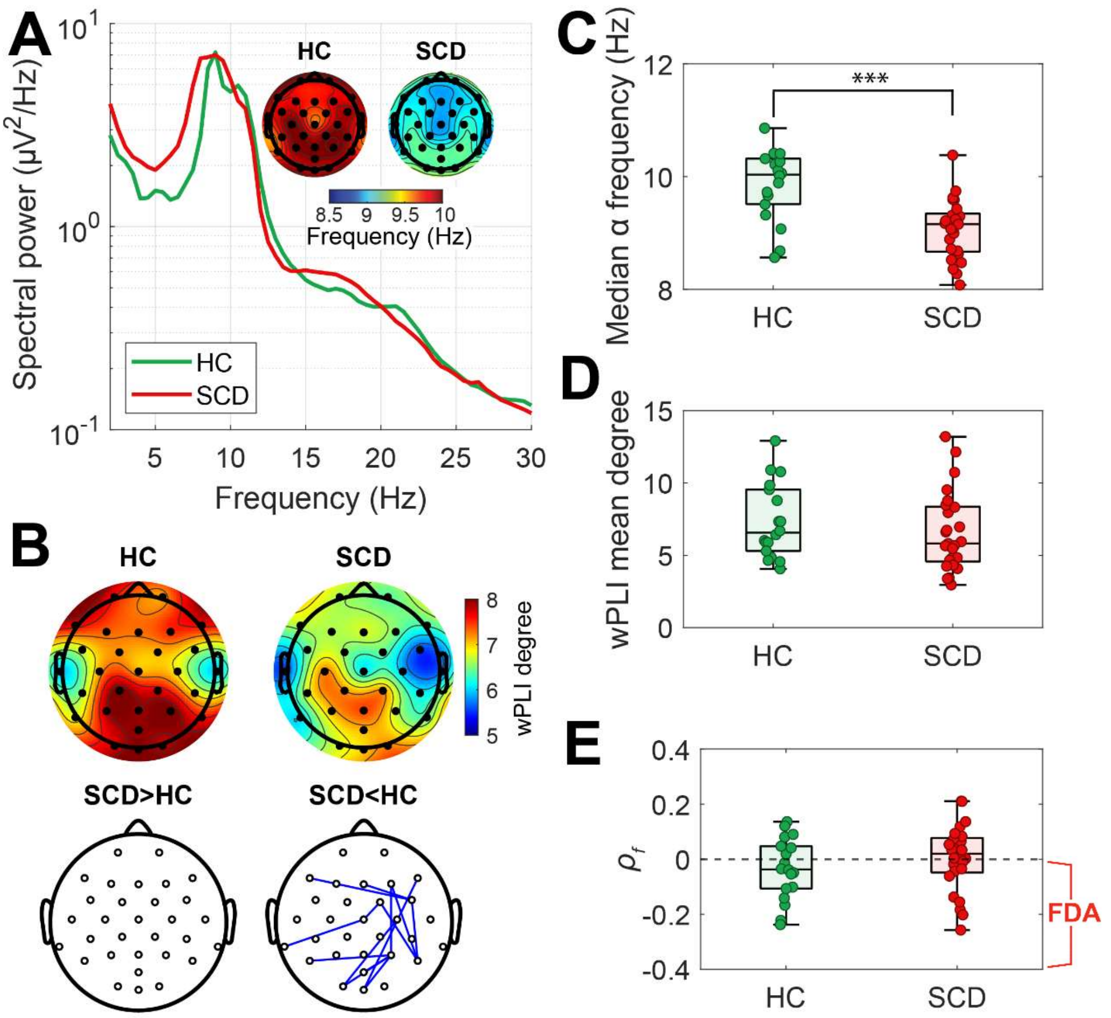

Supplementary Figure 3. Analysis results of EEG under stimulation. A) EEG spectral power and topographic plots showing median alpha (7-13 Hz) frequency. B) Topographic plot displaying wPLI degree and the significant difference of wPLI between HC and SCD. wPLI degree is calculated as the sum of weights attributed to the node. There was no significant difference in wPLI degree. Significantly stronger or weaker pairwise wPLI in SCD was indicated by red and blue lines, respectively. C) Comparison of average median alpha frequencies between the HC and SCD groups ($p<10^{-4}$). D) Mean values of node-wise wPLI degree were not significantly different. E) FDA was not significantly different. ρ_f is Spearman correlation between median alpha frequencies and the average median alpha frequency of the connected nodes in the binarized wPLI network. The boxplots include horizontal lines indicating the 100%, 75%, 50%, 25%, and 0% percentiles. The statistics were derived from a two-sample t-test.

Inclusion Criteria
• Any gender
• 14-17 (Adolescents) and 18-80 (Adults) years old
• Right-handed
• Have been diagnosed with SCD (includes but not limited to SS, SC or other type) and experiencing chronic pain in the past 6 months or vaso-occlusive crisis (VOC) in the past 12 months.
• Subjects with ongoing VOC (or hospitalization during enrollment will not be scheduled for QST and MRI sessions, other scheduled sessions will remain.
• Willing to limit the current and the introduction of any new medications or treatment modalities for control of pain symptoms during the study visits.
• Able to travel to the study site for participating scheduled visits (questionnaires, QST, EEG and MRI) and receive acupuncture treatments up to two times weekly for 5 weeks as scheduled.
• Fluent in English and capable of giving written informed consent.
**Exclusion Criteria**
• Subjects with Covid-19 suspicion or confirmation
• Recent/ongoing alternative pain management with acupuncture or acupuncture-related techniques within the last 6-months.
• Presence of a known coagulation abnormality: Thrombocytopenia (mild thrombocytopenia with a platelets range of 51,000-100,000/ul will be further evaluated for inclusion consideration), or bleeding diathesis that may preclude the safe use of acupuncture.
• Presence of a concurrent autoimmune or inflammatory disease such as rheumatoid arthritis, systemic lupus erythematosus, inflammatory bowel disease, etc. that causes pain or any other chronic pain condition with pain greater than sickle pain.
• Recent (30 days) initiation or dose adjustment of stimulant medications, such as those used to treat ADD/ADHD (e.g., amphetamine/dextroamphetamine [Adderall®], methylphenidate, dextroamphetamine), or the fatigue associated with sleep apnea or shift work (e.g., modafinil).
• Disease/Condition History: Head injury with substantial loss of consciousness, peripheral neuropathy of known cause that interferes with activities of daily living, known non-SCD related Severe psychiatric illnesses (e.g. current schizophrenia, major depression with suicidal ideation), and/or significant visual, motor, or auditory impairment that would interfere with ability to perform study visits-related activities
• Contraindications to MRI scans includes but are not limited to: surgical clips, surgical staples, metal implants, cardiac rhythmic disorders, seizure disorders, and certain metallic dental material will not be scheduled for MRI visits.
• Exclusion for partial QST or MRI scan session: History vascular surgery in lower limbs or current lower limb vascular dysfunction will not receive conditioned pressure pain stimuli in the lower limb.
• Subjects with Worker's Compensation, Workman's Compensation, civil litigation or disability claims pertinent to the subject's sickle disease; current involvement in out-of-court settlements for claims pertinent to the subject's sickle disease; or currently receiving monetary compensation as a result of any of the above.
• Participation of other studies: Concurrent participation in other therapeutic trials with overlapping research purposes.
• Pregnant or nursing.

Supplementary Table 1. Study Eligibility Criteria
